# Structural properties in ruptured mitral chordae tendineae measured by synchrotron-based X-ray phase computed tomography

**DOI:** 10.1107/S1600577523005167

**Published:** 2023-08-18

**Authors:** Yojiro Koda, Takuro Tsukube, Masato Hoshino, Naoto Yagi, Hatsue Ishibashi-Ueda, Kenji Okada

**Affiliations:** a Department of Surgery, Division of Cardiovascular Surgery, Kobe University Hospital, Kobe, Japan; b Division of Cardiovascular Surgery, Japanese Red Cross Kobe Hospital, Kobe, Japan; c Scattering and Imaging Division, Japan Synchrotron Radiation Research Institute, Sayo, Japan; d Department of Pathology, Hokusetsu General Hospital, Takatsuki, Osaka, Japan; RIKEN SPring-8 Center, Japan

**Keywords:** synchrotron-based X-ray phase computed tomography, mitral valve, mitral chorda, mitral chorda rupture, mitral valve structure

## Abstract

The link between the structural properties and the rupturing of chordae tendineae in the mitral valve complex is still unclear. Synchrotron-radiation-based X-ray phase computed tomography (SR-XPCT) imaging is an innovative way to quantitatively analyze three-dimensional morphology. XPCT has been employed in this study to evaluate the chordae tendineae from patients with mitral regurgitation and to analyze structural changes in the ruptured chordae tendineae in patients with this condition. Six ruptured mitral chordae tendineae were obtained during surgical repairs for mitral regurgitation and were fixed with formalin. In addition, 12 healthy chordae tendineae were obtained from autopsies. Employing XPCT (effective pixel size, 3.5 µm; density resolution, 1 mg cm^−3^), the density of the chordae tendineae in each sample was measured. The specimens were subsequently analyzed pathologically. The mean age was 70.2 ± 3.0 in the rupture group and 67.2 ± 14.1 years old in the control group (*p* = 0.4927). All scans of chorda tendineae with SR-XPCT were performed successfully. The mean densities were 1.029 ± 0.004 in the rupture group and 1.085 ± 0.015 g cm^−3^ in the control group (*p* < 0.0001). Density based on SR-XPCT in the ruptured mitral chordae tendineae was significantly lower compared with the healthy chorda tendinea. Histological examination revealed a change in the components of the connective tissues in ruptured chorda tendinea, in accordance with the low density measured by SR-XPCT. SR-XPCT made it possible to measure tissue density in mitral chordae tendineae. Low density in mitral chordae tendineae is associated with a greater fragility in ruptured mitral chordae tendineae.

## Abstract 2

Tissue density measured by synchrotron-based X-ray phase computed tomography is shown to be associated with the fragility of the ruptured mitral chordae tendineae.

## 1. Introduction

Rupture of chorda tendinea (RCT) is the main cause of primary mitral valve regurgitation (MR) (Nishimura *et al.*, 2016 ▸), with mitral valve repair chosen as the first-line treatment for MR caused by RCT. However, even when the anatomy of the mitral valve complex inside the heart has been established, the association with the structural properties and the rupturing of chordae tendineae has not been fully investigated. Therefore, there is a possibility of a recurrence of MR following conventional mitral valve repair (Nishida *et al.*, 2018 ▸; Murashita *et al.*, 2013 ▸).

X-ray phase computed tomography (XPCT) using synchrotron radiation is more sensitive than absorption computed tomography (CT), but it is by no means superior in spatial resolution. Furthermore, the high sensitivity of phase-contrast imaging enables the visualization of small density differences in the biological soft tissue (Margaritondo & Meuli, 2003 ▸; Momose & Fukuda, 1995 ▸). An XPCT using a grating interferometer developed by the Japan Synchrotron Radiation Research Institute (SPring-8) has been used for several soft biological tissues (Hoshino *et al.*, 2012 ▸; Tsukube *et al.*, 2017 ▸). A physical quantity about 1000 times larger than that in the attenuation is involved in the phase shift; thus, in principle, X-ray phase tomography has a high sensitivity for soft tissues (Margaritondo & Meuli, 2003 ▸; Momose & Fukuda, 1995 ▸). The ability to visualize small differences in density and fine details in the structure is attributable to high spatial and density resolution (Tsukube *et al.*, 2017 ▸). This suggests that the application of XPCT to obtain densitometric data for mitral chordal pathology could be useful for investigating their morphological change. This study aimed to use XPCT to investigate structural changes in the chorda tendinea in RCT through detailed visualization and structural quantification.

## 2. Methods

### 2.1. Mitral valve chordae samples

This study was approved by the Japanese Red Cross Kobe Hospital Research Subjects Review Boards (#40, 10/7/2015) and the SPring-8 Proposal Review Committee (2015B1508, 2016A1165, 2017B1163, 2019B1542, 2021B1449), and informed consent was obtained from all patients. The investigation conformed to the principles outlined in the Declaration of Helsinki. Ruptured mitral chordae (*n* = 6) were obtained from patients who had undergone elective mitral valve surgery for MR at the Japanese Red Cross Kobe Hospital. Resected mitral chordae were fixed with 10% buffered formalin, and 3D images were obtained within six months using XPCT (named as the rupture group). The RCT were located on the medial side of the posterior mitral leaflet (P2) in five chordae and on the medial side of the anterior mitral leaflet (A2) in one chorda. In addition, 12 formalin-fixed normal chordae tendineae were obtained from the hearts of cadavers without heart valve disease (named as the control group). The mean age of patients in the rupture group was 70.2 ± 3.0 years (range 65–72 years) and that of patients in the control group was 67.2 ± 14.1 years (range 38–83 years). Patient characteristics are presented in Table 1 ▸.

### 2.2. X-ray phase-contrast tomography imaging

The XPCT system used at the SPring-8 synchrotron radiation facility is based on an X-ray Talbot grating interferometer in the bending-magnet synchrotron beamline (BL20B2), and has been described in detail elsewhere (Hoshino *et al.*, 2016 ▸, 2017 ▸). The main specifications of the grating interferometer used for X-ray phase tomography at the X-ray energy of 18.8 keV are as follows:

(i) G1 as a phase grating – material Ta, pattern thickness 2.1 µm, grating period 10 µm.

(ii) G2 as an absorption grating – material Au, pattern thickness 16.6 µm, grating period 10 µm.

On the other hand, the specifications of another grating interferometer for 20 keV are as follows:

(i) G1 – material Ni, pattern thickness 3.7 µm, grating period 2.4 µm.

(ii) G2 – material Au, pattern thickness 25 µm, grating period 2.4 µm.

Briefly, the system was located 43 m from an X-ray source, and tuned to 18.8 keV or 20 keV by passing it through a silicon double-crystal monochromator. The specimen was embedded in agarose gel and placed in an appropriately sized plastic canister filled with saline solution. Then, the specimen was rotated slowly in the canister using a rotating stage. The examination room was maintained at a constant temperature. A Talbot grating interferometer comprising a phase grating and an absorption grating was placed behind the specimen, and Moiré fringes generated by the interferometer were detected by an X-ray detector. A fringe scanning method was used for phase retrieval where the number of phase stepping was set to five. Phase retrieval was attained using a five-step phase-stepping procedure that involved shifting the absorption grating with a piezo-driven stage. The data were processed by a high-throughput system to create 3D images. The specifications of the system were as follows: field of view, 13.3 mm (H) × 5.0 mm (V) with the voxel size of 6.5 µm or 14.1 mm (H) × 5.0 mm (V) with the voxel size of 3.4 µm; target density range, 0.9–1.2 g cm^−3^; density resolution, 1 mg cm^−3^; exposure time, 100–400 ms per image for static observations. Due to the improvement of the setup of the grating interferometer, the energy used and the specification of the grating interferometer optimized for that differ depending on the timing of the measurement. However, for all measurement conditions, the calculated density values were calibrated by measuring a standard sample of known density, in this case a salt solution with different concentrations. We used saline solutions of three different densities (1.026 g cm^−3^, 1.053 g cm^−3^ and 1.102 g cm^−3^) to determine the ratio of the experimental values to the theoretical values. We have confirmed that the plot of the experimental values compared with the theoretical values is linear when the densities are changed. In cases involving the observation of a chorda tendinea longer than the effective vertical field view, the specimen was scanned step-by-step vertically, with a stepping amount of 4 mm per tomographic scan to observe its whole structure. A conventional filtered back-projection (FPB) method with a Chesler filter was used to calculate the reconstruction of the cross-sectional images. Any filters or image processing to remove the ring artifact were not applied. The phase image was calculated by integrating the differential phase image in one direction. The following relation between the density difference (Δρ) and the refractive index difference (Δδ) from the background was used to calculate the density,

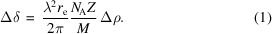

Here, λ is the X-ray wavelength, *r*_e_ is the classical electron radius, *N*_A_ is Avogadro’s constant, and *Z*/*M* is the ratio of the number of electrons to the molecular weight of the sample, here set to 0.55. Then, the density was calculated from ρ = ρ_BG_ + Δρ, where ρ_BG_ is the density of the background. In X-ray phase tomography, the sample was measured in a dedicated plastic container filled with normal saline. Therefore, the density of the background ρ_BG_ corresponds to that of normal saline in this study.The reconstructed volumes were converted into 16-bit TIFF images of each tomography slice with custom-made software. Further image processing and analysis of the tomography data was performed with *ImageJ* (https://rsbweb.nih.gov/ij/index.html) (Momose & Fukuda, 1995 ▸; Yagi *et al.*, 1999 ▸).

Morphological differences were quantitatively evaluated by estimating the mean mass density within a region of interest in the chordae tendinea for each formalin-fixed sample. Each region of interest was a cross-sectional area × 100 voxels as depth. The mass density of the chordae tendinea was measured at around the papillary muscle attachment site in the normal chordae tendinea as control. In ruptured chordae tendinea, the density was measured at two portions, including the ruptured portion located near the papillary muscle attachment site and the non-ruptured portion near the mitral leaflets.

### 2.3. Histological examinations

After XPCT imaging was completed, the samples were stored in 10% formalin and sectioned transversely. Thereafter, the sections of each specimen were stained with hematoxylin and eosin, and Sirius red. The Sirius red stains were used to detect elastic fibers and collagen fibers. Tenomodulin was identified by immunostaining with an anti-tenomodulin antibody (Sigma-Aldrich, St Louis, MO, USA) and used to detect the healing process in chorda tendinea (Shukunami *et al.*, 2016 ▸). The histological examination focused on the identification restoration process, including inflammation and abnormalities in tissue components such as collagen and elastic fibers. Moreover, to evaluate the contribution of collagen fibers to densitometric changes in the chorda tendinea, the occupation ratio of collagen fibers was determined in the sample using *ImageJ*. The occupation ratio was defined as the area occupied by collagenous components as a percentage of the selected area.

### 2.4. Statistical analysis

All statistical tests were conducted using the JMP statistical software (SAS Institute, Cary, NC, USA). Students’ t-tests were employed to compare the densities in the chorda tendinea. The significance was set at *P* < 0.05 for all the statistical analyses. Data are presented as mean ± standard deviation values.

## 3. Results

### 3.1. Representative XPCT Images

**Figure 1:**
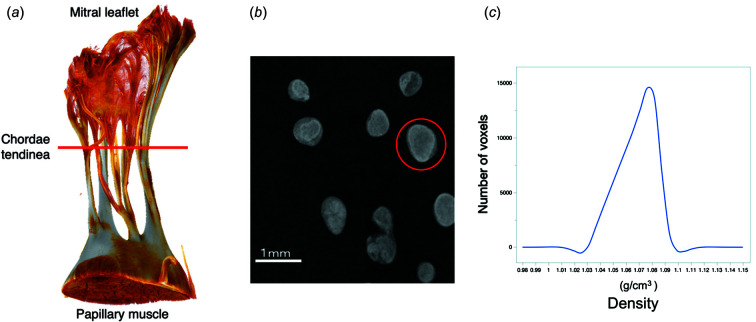
(*a*) Clear and detailed three-dimensional imaging of a chordae tendinea from the papillary muscle to the leaflet of a normal mitral valve obtained by synchrotron-based X-ray phase computed tomography. (*b*) A selected marginal chorda tendinea (red circle) in an axial image after the imaging process. (*c*) Calculating density from TIFF images of each tomography slice of the selected samples.

Fig. 1 ▸(*a*) clearly demonstrates that XPCT can provide a clear and detailed three-dimensional image of a chordae tendinea from the papillary muscle to the leaflet of a normal mitral valve. After XPCT imaging, the target marginal chorda tendinea was selected in an axial image [Fig. 1 ▸(*b*)], and, as explained in the *Methods* section[Sec sec2], its density was obtained from TIFF images of each tomography slice sample. The tissue density in the chordae tendinea of a normal mitral valve was almost homogeneous, with a single peak in the density histogram at approximately 1.0816 g cm^−3^ [Fig. 1 ▸(*c*)].

### 3.2. Quantitative assessment of the chorda tendinea

**Figure 2:**
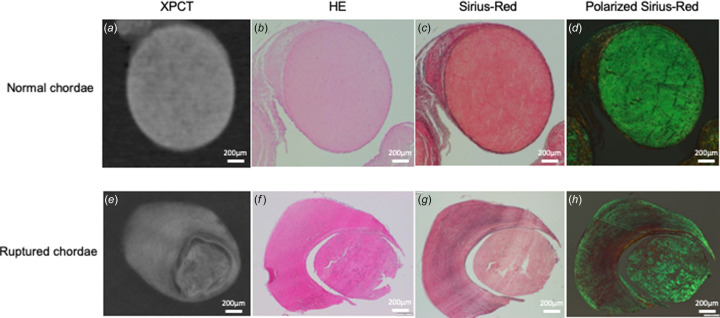
An axial image of the XPCT (*a*, *e*), HE (*b*, *f*), Sirius red (*c*, *g*), and polarization of the Sirius red (*d*, *h*) in a normal chorda (upper row) and ruptured chorda (lower row). The mass density was 1.092 g cm^−3^ in the normal chorda (*a*) and 1.034 g cm^−3^ in the ruptured chordae (*e*). The differences in the mass density between the normal and ruptured chorda were consistent with the distribution of collagen fibers observed in the associated histological samples. XPCT – X-ray phase computed tomography; HE – hematoxylin-eosin.

**Figure 3:**
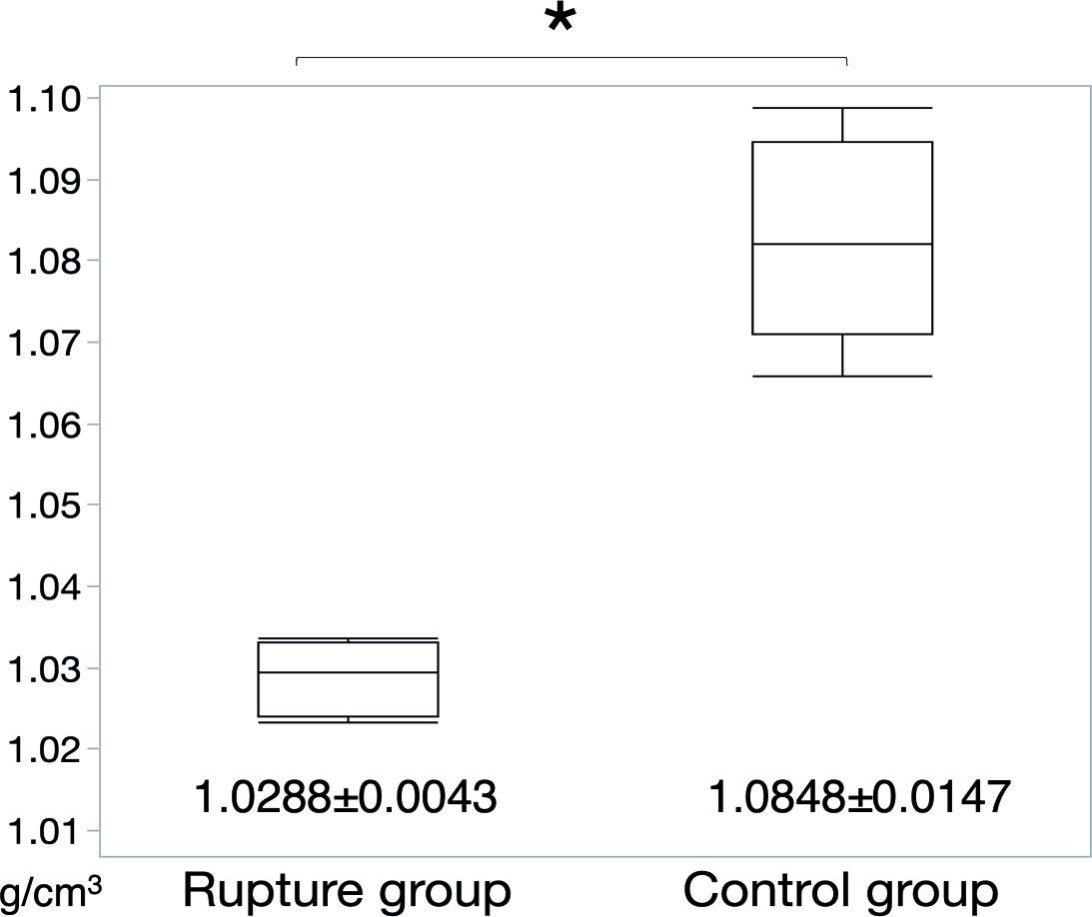
Comparison of the mass density between the chorda rupture group and control group. The mass density based on the synchrotron-based X-ray phase computed tomography in the ruptured mitral chordae tendineae was significantly lower compared with normal chorda tendinea. *p < 0.05.

**Figure 4:**
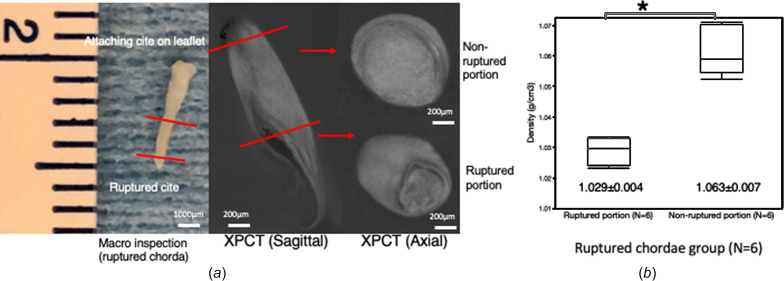
The distribution of the mass density in the ruptured chordae tendinea. (*a*) Analyzing the mass density of the ruptured portion and non-ruptured portion in the ruptured chordae tendineae. (*b*) Comparison of the mass density between the ruptured portion and non-ruptured portion in the ruptured chordae tendineae. There was a significant difference in the density of the ruptured portion and non-ruptured portion. *p < 0.05.

**Figure 5:**
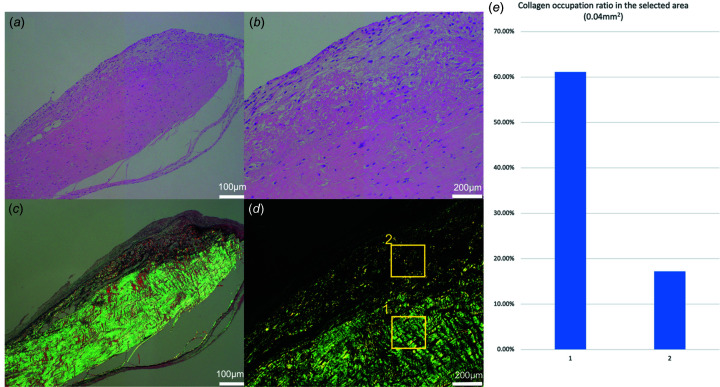
Sagittal section of the ruptured chordae stained with hematoxylin-eosin (HE) and polarization of the Sirius red (SR) staining, and the collagen occupation ratio. (*a*) There was a non-longitudinal abnormal interstitial tissue layer consistent with the pathological cross-sectional findings (HE). (*b*) Inflammatory infiltration was not seen in the entire layer, including the abnormal interstitial tissue and longitudinal collagen bundle (HE). (*c*) SR staining revealed that the non-longitudinal abnormal connective tissue layer included elastic fibers (black color lesion) and collagen fibers (yellow color lesion; type I collagen, green color lesion; type III collagen). (*d*) The collagen occupation ratio was calculated in the two selected areas, including an area of abundant abnormal connective tissue (yellow rectangle #2) and an area of normal collagen bundle (yellow rectangle #1) (SR staining). (*e*) The collagen occupation ratio was 61.1% in the area of normal collagen bundle and 17.2% in the area of abundant abnormal connective tissue.

To quantitatively evaluate morphological differences, the mass densities of the chorda tendinea were estimated in each sample. In the control group (*n* = 12), the mean density of the chorda tendinea was 1.085 ± 0.015 g cm^−3^, with no significant variation within the chorda. These findings were consistent with the histological findings for the same region, wherein uniform high-density collagenous fibers were observed in the media (Fig. 2 ▸). These findings may show that the density of the chorda tendinea is homogeneous, providing the chorda with a stable structure. In the rupture group, the mean density of the ruptured portion of the chorda tendinea was 1.029 ± 0.004 g cm^−3^, significantly lower than the mass density for normal mitral chordae (*p* < 0.0001) (Fig. 3 ▸). The differences in the mass density between the normal and the ruptured chorda were consistent with the distribution of collagenous fibers observed in the associated histological samples (Fig. 2 ▸). The distribution of mass density in the ruptured chordae, *i.e.* not including portions that were not ruptured, was measured by analyzing each ruptured chordae tendinea [Fig. 4 ▸(*a*)] at six points. There was a significant difference in the density of the ruptured portion and non-ruptured portion (1.029 ± 0.004 versus 1.063 ± 0.007 g cm^−3^, respectively; *p* = 0.005) [Fig. 4 ▸(*b*)]. Figs. 5 ▸(*a*) and 5(*b*) show formalin-fixed specimens of the ruptured chordae, and XPCT image and mass densities of ruptured and non-ruptured portions. The differences in the mass density were consistent with the histological findings, demonstrating that pathogenesis of RCT might be a local event rather than occurring along the entire length of the chordae tendinea.

**Figure 6:**
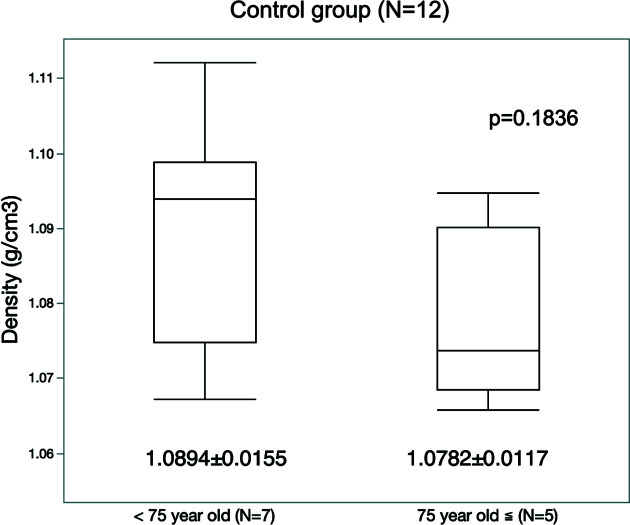
Comparison of the mass density between samples aged 75 years or older (aged group; *n* = 5) and samples less than 75 years old (non-aged group; *n* = 7) in the control group. The mass density of the chordae tendinea was slightly lower in the aged group, but there was no statistically significant difference between the two groups.

In addition, to assess the effect of aging on the mass density of mitral chorda, a comparison was made of the density of chordae tendinea from normal mitral valve between samples from people aged 75 years or older (aged group; *n* = 5) and samples from those aged below 75 years (non-aged group; *n* = 7) in the control group. The mass density of the chordae tendinea was 1.078 ± 0.012 g cm^−3^ in the aged group and 1.089 ± 0.016 g cm^−3^ in the non-aged group, *i.e.* slightly lower in the aged group. However, there was no statistically significant difference between the two groups (*p* = 0.1836) (Fig. 6 ▸).

### 3.3. How the collagen in the obtained CT looks compared with histology

Images from histological examinations of normal and ruptured chordae tendinea are shown in Fig. 2 ▸. In the normal chordae, highly dense collagen fibers occupied almost all cross-sectional surfaces and the constituted collagen bundle [Figs. 2 ▸(*b*) and 2(*c*)]. In the Sirius red staining, black lesions found on subendothelial small interstitial tissues indicated elastic fibers [Fig. 2 ▸(*c*)]. Polarization of the synchrotron radiation staining demonstrated that collagen fiber (type III), stained in green, occupied most of the collagen bundle, along with a small interstitial tissue area beneath the endothelial layer [Fig. 2 ▸(*d*)]. By contrast, ruptured chorda stained with hematoxylin and eosin showed increased abnormal interstitial tissue growth surrounding the collagen bundle [Fig. 2 ▸(*e*)]. Synchrotron radiation staining of the ruptured chordae showed an increase in abnormal interstitial tissue, with more elastic fibers and fewer collagen fibers compared with the adjacent collagen bundle [Figs. 2 ▸(*f*) and 2(*h*)]. The obtained CT images reflected the differences of constituent elastic and collagen fibers in normal and ruptured chordae tendinea. Normal chorda tendinea showed a uniform high signal indicating collagen bundles, while, in ruptured chorda, core collagen was surrounded by a signal reflecting abnormal interstitial tissue, like annual rings on a tree [Figs. 2 ▸(*a*) and 2(*e*)].

### 3.4. The difference of collagen occupation ratio in ruptured chordae tendinea in histology

**Figure 7:**
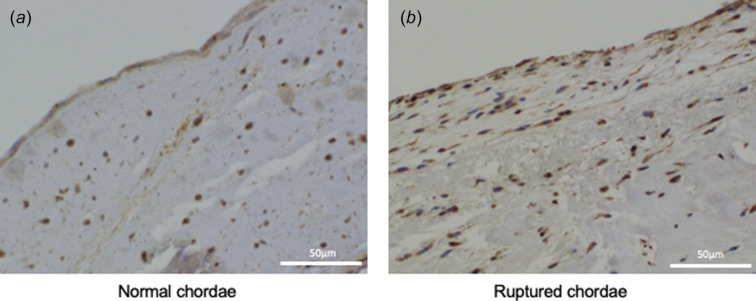
Immunofluorescence staining for tenomodulin. Tenomodulin positive cells were rare and localized in interstitial tissues under the endothelial cell layer (*a*), whereas, in the ruptured chordae, tenomodulin positive scattered cells were often found to exist in the region of increased abnormal connective tissues (*b*).

In the sagittal section of the ruptured chordae stained with hematoxylin and eosin, a non-longitudinal abnormal interstitial tissue layer consistent with the pathological cross-sectional findings [Fig. 5 ▸(*a*)] was observed. Inflammatory infiltration was not seen in the entire layer, including the abnormal interstitial tissue and longitudinal collagen bundle [Fig. 5 ▸(*b*)]. Polarization of the Sirius red staining revealed that the non-longitudinal abnormal connective tissue layer included elastic fibers (black color lesion) and collagenous fibers (yellow color lesion; type I collagen, green color lesion; type III collagen) [Fig. 5 ▸(*c*)]. To quantify the difference in distribution of collagen fibers, the collagen occupation ratio was calculated in two selected areas, including an area of abundant abnormal connective tissue [#2 in Fig. 5 ▸(*d*)] and an area of normal collagen bundle [#1 in Fig. 5 ▸(*d*)]. The collagen occupation ratio was found to be 61.1% in the area with a normal collagen bundle and 17.2% in the area with abundant abnormal connective tissue [Fig. 5 ▸(*e*)]. Moreover, immuno­staining with anti-tenomodulin (Tnmd) antibody was performed to detect the healing process in the chorda tendinea. In the normal chordae, Tnmd positive cells were extremely rare and localized in interstitial tissues beneath the endothelial cell layer [Fig. 7 ▸(*a*)]. In the ruptured chordae, however, Tnmd positive scattered cells were often found in the region of increased abnormal connective tissues [Fig. 7 ▸(*b*)], which was viewed as an explanation for any healing processes that had taken place in the ruptured chordae tendinea.

## 4. Discussion

Synchrotron-radiation-based XPCT with Talbot interferometry (Momose *et al.*, 2003 ▸) has shown promise as a means of visualizing the microstructure of several biological samples (Momose, 2005 ▸) and tissues, including the lungs (Momose & Fukuda, 1995 ▸), brain (Yagi *et al.*, 1999 ▸), eyes (Hoshino *et al.*, 2011 ▸), aorta (Zanette *et al.*, 2012 ▸) and heart (Kaneko *et al.*, 2017 ▸). In principle, synchrotron-radiation-based XPCT is about 1000 times more sensitive compared with conventional X-ray absorption-contrast techniques (Momose & Fukuda, 1995 ▸). It has three major advantages: it provides high-resolution and 3D images without destroying/altering the properties of specimens; it enables the visualization of tissue microstructures and mass density without the need for a contrast agent; and it allows quantitative analyses of the densities of the microstructures of various soft tissues, facilitating quantitative comparisons among many different types of soft tissues (Momose & Fukuda, 1995 ▸; Hoshino *et al.*, 2011 ▸, 2012 ▸).

The RCT is a common cause of primary mitral regurgitation, and mitral valve repair has been an established procedure for MR (Nishimura *et al.*, 2016 ▸). However, the causes of RCT and structural properties of the mitral chordae tendinea have not been fully elucidated. In this study, we performed a quantitative comparison among chordae tendinea that revealed differences in the microstructure of the chorda tendinea between specimens from patients with MR due to RCT and those with a normal mitral valve.

The tissue density in the chordae tendinea of the normal mitral valve was almost homogeneous, with a single peak in the density histogram of approximately 1.085 g cm^−3^. To clarify the effect of aging on the tissue density of the chordae tendinea, the chordae tendinea of the normal mitral valve was divided into two groups and analyzed. There was no statistically significant difference between the tissue density of the chordae tendinea belonging to the aged group (75 years or older) and non-aged group (below 75 years old), even though the density in the younger group tended to be slightly lower. Although the aged mitral chordae tendinea has been reported to be much stiffer and less extensible than younger chordae (Zuo *et al.*, 2016 ▸), our results may explain that aging alone may not be a sufficient cause of RCT.

It is worth noting that the ruptured portion of the ruptured chordae tendineae of patients with MR showed a significantly lower tissue density in the chordae tendineae (1.029 ± 0.004 g cm^−3^) when compared with tissue density of the non-ruptured portion of the ruptured chordae tendinea, or with that of the chordae tendinea of the normal mitral valve. The differences in the tissue density between the ruptured portion and non-ruptured portion were consistent with the distribution of collagenous fibers observed in the associated histological samples.

A human mitral chorda tendinea comprises an endothelium cell layer, a small number of elastic fibers, and mostly fibrillary collagens (collagen type I and III) (Akhtar *et al.*, 1998 ▸; Stolinski, 1996 ▸). Collagen fibers are the main constituent of the collagen bundle and maintain the strength of the chorda tendinea (Millington-Sanders *et al.*, 1998 ▸; Icardo & Colvee, 1995 ▸). By contrast, elastin fibers surrounding the collagen bundle appear as a mesh-like structure that assist in collagen stretching and contraction, and also act as a barrier to physical stimulation between the collagen and the endothelium (Millington-Sanders *et al.*, 1998 ▸; Fenoglio *et al.*, 1972 ▸). However, elastin fibers rarely contribute to the mechanical properties of chordae tendinea (Lim & Boughner, 1976 ▸). A histological examination of the ruptured chordae tendineae in this study revealed a lower proportion of collagen fibers, a layer of unorganized non-longitudinal tissue between the endothelial cell layer, and an organized longitudinal collagen bundle layer, all of which could be consistent with the connective tissue cushion reported by the previous report (Millington-Sanders *et al.*, 1998 ▸). Given that the connective tissue cushion does not contribute to structural strength as effectively as original elastic fibers, our findings suggested that the strength of the chorda should be reduced (Millington-Sanders *et al.*, 1998 ▸). The obtained CT images showed a uniform high signal in cross-sectional view in normal chordae tendinea, whereas the ruptured chordae tendinea were characterized by the signal of the core cord-like structure and the concentric structures surrounding it. As a result, the obtained CT images were able to detect structural changes in the core collagen surrounded by abnormal interstitial tissues in the ruptured chordae tendinea, which findings are well accorded with pathological findings. Tenomodulin is a type II transmembrane surface molecule (Shi *et al.*, 2017 ▸) mainly expressed in avascular tissues, particularly in dense connective tissue such as tendon/ligament, heart valve/chorda and eye (Oshima *et al.*, 2003 ▸; Kimura *et al.*, 2008 ▸). As Tnmd possesses anti-angiogenesis that maintains avascular tissue – and without inflammatory cells or abnormal connective tissues inducing weakness in the histological strength – Tnmd expression is important in maintaining histological strength in the avascular tendon or ligament tissue. The other roles of Tnmd include maturing tenocyte during the restoration process (Tokunaga *et al.*, 2015 ▸), and Tnmd overexpression favors tenogenic lineage differentiation (Shi *et al.*, 2017 ▸). In our study, increased Tnmd expression was observed in increased abnormal subendothelial connective tissue in ruptured chordae tendinea compared with normal chordae tendinea. These findings could suggest that increased abnormal subendothelial connective tissue in ruptured chordae tendineae is reflected in the chordae during the healing process as lower histological strength, leading eventually to a tear in the chordae tendinea.

Although mitral valvuloplasty is a well established procedure for MR (Lazam *et al.*, 2017 ▸), there have also been reports of recurrent MR requiring repeated mitral valve surgery after an initial mitral valvuloplasty (Nishida *et al.*, 2018 ▸; Murashita *et al.*, 2013 ▸). In this study, we observed the structural properties of ruptured chorda tendineae by evaluating tissue density. In cases of an advanced modality, which could visualize changes in density or associated changes in collagen fibers during open heart surgery or preoperative evaluations, mitral valvuloplasty might be applied clinically, and prophylactic techniques to prevent chordal elongation or rupture after surgical repair could be introduced.

Further structural and molecular understanding of mitral valve pathology and technological advances is required to improve the outcomes of mitral valve surgery.

The presented study included several limitations. First, at 18 in total, the sample size was relatively small. It should be noted, however, that there are few reports that analyze the structural properties of mitral chordae tendineae in human specimens aside from this study. Second, the RCT group in this study included chorda tendinea with attached anterior and posterior leaflets. Ideally, chorda tendinea should be unified only for anterior or posterior leaflets, although it has been reported that there is no significant viscoelastic difference between chorda tendinea for anterior and posterior leaflets in animal durability tests (Chen *et al.*, 2020 ▸). Third, ruptured chordae tendinea investigated in this study were collected from patients with chronic MR due to RCT. Therefore, in terms of outcomes, it is difficult to clarify whether our findings were the cause or effect of RCT of the mitral valve. Further studies using the acute phase RCT model will help facilitate a solution to this problem.

## 5. Conclusion

Synchrotron-radiation-based XPCT made it possible to measure tissue density in mitral chordae tendineae. The homogeneous density of the normal chorda tendinea is consistent with highly dense collagen fibers in the collagen bundle, and a localized low density of the ruptured portion of the ruptured chorda may reflect a healing process of a previous injury in the chordae tendinea resulting in structural alterations. Therefore, low density in mitral chordae tendineae is associated with the fragility of the ruptured chordae tendineae.

## Figures and Tables

**Table 1:** Patient characteristics

	Ruptured chordae group	Control group
Number of chordae	6	12
Age (years)	70.2 ± 3.0 (65–72)	67.2 ± 14.1 (38–83)
Cardiac disease	Mitral regurgitation	None
Location of RCT		
A2†	1	N/A
P2‡	5	N/A

† Medial side of the anterior mitral leaflet.

‡ Medial side of the posterior mitral leaflet.
